# Suppressive effects of pterostilbene on human cytomegalovirus (HCMV) infection and HCMV-induced cellular senescence

**DOI:** 10.1186/s12985-022-01954-4

**Published:** 2022-12-23

**Authors:** Sanying Wang, Xuqiang Zhou, Xinyue He, Shushu Ma, Chuan Sun, Jing Zhang, Xiaogang Xu, Weihua Jin, Jin Yan, Ping Lin, Genxiang Mao

**Affiliations:** 1 Zhejiang Provincial Key Lab of Geriatrics and Geriatrics Institute of Zhejiang Province, Department of Geriatrics, Zhejiang Hospital, Hangzhou, 310030 People’s Republic of China; 2 College of Life Sciences, Zhejiang Chinese Medical University, Hangzhou, 310053 People’s Republic of China; 3 College of Biotechnology and Bioengineering, Zhejiang University of Technology, Hangzhou, 310014 People’s Republic of China; 4 Geriatric Department of the 3rd Hospital of Hangzhou, 310009 Hangzhou, People’s Republic of China

**Keywords:** Pterostilbene, HCMV, Cellular senescence, Reactive oxygen species

## Abstract

**Background:**

Human cytomegalovirus (HCMV), a member of the β-herpesvirus family, causes the establishment of a latent infection that persists throughout the life of the host and can be reactivated when immunity is weakened. To date, there is no vaccine to prevent HCMV infection, and clinically approved drugs target the stage of viral replication and have obvious adverse reactions. Thus, development of novel therapeutics is urgently needed.

**Methods:**

In the current study, we identified a naturally occurring pterostilbene that inhibits HCMV Towne strain replication in human diploid fibroblast WI-38 cells through Western blotting, qPCR, indirect immunofluorescence assay, tissue culture infective dose assays. The time-of-addition experiment was carried out to identify the stage at which pterostilbene acted. Finally, the changes of cellular senescence biomarkers and reactive oxygen species production brought by pterostilbene supplementation were used to partly elucidate the mechanism of anti-HCMV activity.

**Results:**

Our findings revealed that pterostilbene prevented lytic cytopathic changes, inhibited the expression of viral proteins, suppressed the replication of HCMV DNA, and significantly reduced the viral titre in WI-38 cells. Furthermore, our data showed that pterostilbene predominantly acted after virus cell entry and membrane fusion. The half-maximal inhibitory concentration was determined to be 1.315 μM and the selectivity index of pterostilbene was calculated as 26.73. Moreover, cell senescence induced by HCMV infection was suppressed by pterostilbene supplementation, as shown by a decline in senescence-associated β-galactosidase activity, decreased production of reactive oxygen species and reduced expression of p16, p21 and p53, which are considered biomarkers of cellular senescence.

**Conclusion:**

Together, our findings identify pterostilbene as a novel anti-HCMV agent that may prove useful in the treatment of HCMV replication.

## Introduction

Human cytomegalovirus (HCMV) is a β-herpesvirus with a 235 kb double-stranded DNA (dsDNA) genome that encodes at least 165 proteins [1]. HCMV infection is common worldwide. The proportion of IgG positivity in adults is approximately 60% in developed countries and exceeds 90% in many developing countries [2]. HCMV infection in immunocompetent individuals is asymptomatic and ubiquitous; however, infection with HCMV is strongly associated with severe morbidity and mortality in immunocompromised settings, including transplantation and HIV-positive individuals [3]. Infection with HCMV is a major cause of microcephaly, cognitive disabilities, vision loss, sensorineural hearing loss, and vascular disease [4, 5]. Currently, despite the urgent need, HCMV vaccines are not available, and progress towards this target has been slow, although there are numerous candidate vaccines in development [6, 7]. Four drugs have been used clinically for HCMV infection: cidofovir (CDV), ganciclovir (GCV), foscarnet (FOS) and, recently, letermovir (LMV) [8]. Of these, GCV, FOS and CDV have similar antiviral mechanisms in that they mainly target viral DNA polymerase and inhibit the synthesis of viral DNA [8], but they do not prevent multiple signal events during the other infection cycle [9]. LTV targets the viral terminase complex and inhibits the packing of DNA into capsids [8]. However, the emergence of drug-resistant strains and refractory HCMV strains [10], along with the occurrence of severe side effects, have reduced the effectiveness of these treatments [8]. HCMV can infect a wide variety of cell types, including fibroblasts, epithelial cells, macrophages, endothelial cells, dendritic cells and smooth muscle cells [11]. For these reasons, it is necessary to develop new alternative drugs for HCMV infection with modified safety, efficacy, and resistance profiles [8].

**Fig. 1:**
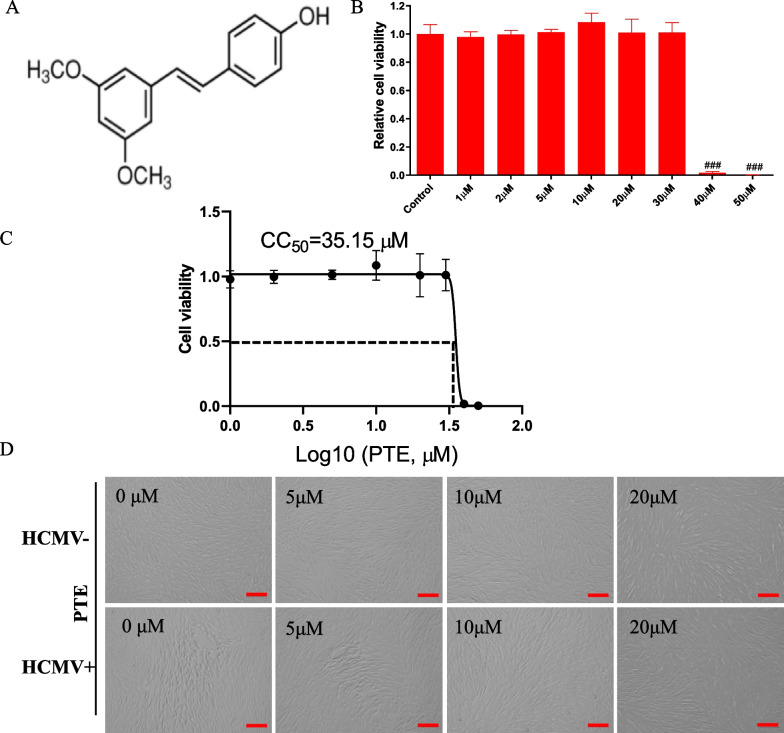
**A** Chemical structure of PTE. **B** The effect of PTE on cell viability was assayed at 5 days post-treatment. WI-38 cells were treated with PTE at the indicated concentrations or vehicle. Cell viability was analysed by CCK-8 assays. **C** The falf-maximal cytotoxicity concentration (CC50) was determined at 35.15 μM based on the results of the cell viability assay according to nonlinear trajectory analysis using GraphPad Prism. **D** Effects of PTE on morphological changes with or without HCMV infection. WI-38 cells were infected with HCMV at an MOI of 0.5, and typical CPE were observed at 5 dpi. In the PTE treatment group, WI-38 cells were pretreated with PTE at the indicated concentrations 2 h before HCMV infection. ^###^*P* < 0.001 versus the control, bar: 200 μm

Pterostilbene (trans-3,5-dimethoxy-40-hydroxystilbene, PTE), belonging to a group called stilbenes, is a constituent of *Pterocarpus marsupium* and was isolated in 1940 [12]. Its structure was confirmed and analysed by synthesis in 1941 [13]. Structurally, PTE is a dimethyl ether analogue of resveratrol (RES), which is a polyphenolic natural product found in some plants, such as grapes, plums, and peanuts [14]. Owing to the increased lipophilicity of its two methoxyl groups (Fig. 1A), PTE has higher intestinal absorption, bioavailability and biological activities, such as anticancer, antioxidant, anti-inflammatory and analgesic activites than RES [15–17]. Moreover, recent studies have suggested that PTE may improve risk factors associated with fatty liver disease, diabetes, cardiovascular disease, and Alzheimer's disease [18]. PTE can also attenuate fructose-induced myocardial fibrosis by inhibiting ROS-driven Pitx2c/miR-15b pathway [19].

Drug repurposing is a promising, convenient and cost- and time-effective antiviral strategy for the development of new therapeutic solutions [20]. The current novel pneumonia caused by severe acute respiratory syndrome coronavirus-2 (SARS-CoV-2) offers a good example of how this approach could be beneficial in the treatment of viral infection [21, 22]. There are many reports about the antiviral effect of RES [23] including SARS-CoV-2 [24–26], but to date, antiviral studies of PTE are mainly related to HIV [27, 28] and SARS-CoV-2 [26]. In this study, we describe for the first time the role and regulatory mechanism of PTE in HCMV infection in vitro.

## Methods

### Compounds, cells, viruses, reagents, and antibodies

Foscarnet (FOS) was purchased from Sigma-Aldrich (St. Louis, MO, USA). PTE was purchased from Shanghai Standard Technology Co., Ltd. (Shanghai, China) and dissolved in vehicle (DMSO) for a stock solution of 10 mM. Human diploid fibroblasts (WI-38 cells) and HCMV (Towne strain) were obtained from the American Tissue Culture Collection (ATCC, catalogue no. VR-977, Manassas, VA, USA). The QIAamp DNA mini kit was obtained from Qiagen (Hilden, Germany). Dulbecco’s modified Eagle’s medium (DMEM), foetal bovine serum (FBS), 100 × penicillin-streptomycin solution, and 0.25% trypsin-EDTA were purchased from Gibco-BRL Life Technologies (Grand Island, NY, USA). Mouse monoclonal antibodies against immediate-early (IE1/2, catalogue no. P1215) and early protein (UL44, catalogue no. CA006-100) of HCMV were obtained from Virusys Corporation (Randallstown, MD, USA). Anti-p16^INK4a^ (catalogue no. 92803S), anti-p21 (catalogue no. 2947S), and anti-p53 (catalogue no. 2527S) primary rabbit antibodies were purchased from Cell Signaling Technology (Boston, MA, USA). The Senescence-associated β-galactosidase (SA-β-Gal) Staining Kit and polyvinylidene fluoride (PVDF) membrane were purchased from Cell Signaling Technology (Boston, MA, USA) and Bio-Rad Laboratories (Hercules, CA, USA), respectively. The 2 × Universal SYBR Green Fast qPCR Mix was obtained from Abclonal Technology (Wuhan, China). Cell lysis buffer for Western blotting, 5 × SDS‒PAGE protein sample loading buffer, Cell Counting Kit-8 and Reactive Oxygen Species (ROS) Assay Kit were all obtained from Beyotime Institute of Biotechnology (Shanghai, China). 10 × Tris-glycine SDS‒PAGE running buffer and 10 × Tris-glycine transfer buffer were obtained from Sangon Biotech (Shanghai, China). HRP-conjugated goat anti-mouse IgG, HRP-conjugated goat anti-rabbit IgG, and FITC-conjugated goat anti-mouse IgG were all purchased from Hangzhou Huaan Biotechnology (Hangzhou, China).

### HCMV infection and PTE treatment

WI-38 cells were cultured in DMEM with 10% FBS and penicillin-streptomycin solution. WI-38 cells were considered young at a population doubling (PD) of 30 or less, whereas cells at PD 50 or higher were defined as replicative senescent. All experiments in this study were performed using young cells ranging from 25 to 30 PD.

Stocks of the Towne strain of HCMV were routinely prepared as previously mentioned [29–31]. Before virus infection, the culture medium was replaced with 0.2% FBS for 48 h to synchronize cells in the G_0_ phase. The cells were mock-infected or infected with HCMV (MOI = 0.5) for 2 h at 37 °C. Then, the inoculum was discarded, and the cells were washed and incubated with fresh culture medium with 0.2% FBS in the absence or in the presence of PTE. FOS (200 µg/mL) was used as a positive-drug control. The cell samples were harvested at the indicated times. In the PTE and FOS treatment groups, WI-38 cells were pretreated with PTE and FOS 2 h before virus infection.

### CCK-8 assay

The effect of PTE on cell viability was determined by CCK-8 assays. Briefly, WI-38 cells were seeded in 96-well plates at a density of 5 × 10^3^ cells/well and incubated at 37 °C. Subsequently, culture medium containing different concentrations of PTE (1 μM, 2 μM, 5 μM, 10 μM, 20 μM, 50 μM, 100 μM) was added and incubated for 5 days. At the predetermined time, 10 μL of CCK-8 was added to each well. After incubation for 1–2 h at 37 °C, absorbance was measured using a Thermo Scientific microplate reader (Multiskan ™ FC) at 450 nm according to the standard protocol. The vehicle (DMSO) group was used as the control. Each group was assayed in triplicates (n = 3).

### Time-of-addition experiments

For the “Entry” assay, PTE or FOS was added to the cells for 2 h, followed by HCMV inoculation for 1 h. Then, we removed the culture medium containing HCMV and the drug and washed the cells three times with preheated PBS. Finally, DMEM with 0.2% FBS was added to each well and maintained until sample collection. For the “Post-Entry” assay, first, HCMV was inoculated into WI-38 cells for 1 h at 37 °C. Then, we discarded the virus and washed the cells thrice with preheated PBS to remove unbound virus. Finally, DMEM with 0.2% FBS containing PTE or FOS was added to each well and maintained until sample collection. For all the experimental groups, the concentrations of PTE and FOS were 10 µM and 200 µg/mL, respectively. WI-38 cells were infected with HCMV (MOI = 5) and harvested 3 dpi. The HCMV DNA copy numbe and viral proteins in the infected cells were quantified by qPCR and Western blotting.

### Western blotting

The cell samples were prepared as described above and lysed with cell lysis buffer. Then, 50 μg of protein extract was subjected to SDS‒PAGE and transferred to PVDF membranes. Blocking was performed with 5% nonfat dry milk for 1 h at room temperature, and the membranes were incubated with the corresponding antibodies mentioned above (IE1/2-1:500 dilution, UL44-1:10000 dilution, p16-1:1000 dilution, p21-1:1000 dilution, p53-1:1000 dilution). HCMV-infected alone, and FOS-pretreated groups were used as controls.

### qPCR

The cell samples were prepared as described above and the cell culture supernatant was discarded. Then, the cells were washed with PBS and harvested with 0.25% trypsin-EDTA. HCMV DNA was extracted with a QIAamp DNA Mini Kit according to the manufacturers’ instructions. The copy number of HCMV DNA was evaluated using qPCR assay with 2 × Universal SYBR Green Fast qPCR Mix [30, 31]. The HCMV *UL 123*, *pp150* and *GAPDH* primers used in this study were as follows: HCMV *UL123*-forward 5′-TCTGCCAGGACATCTTTCTC-3′ and reverse 5′-GTGACCAAGGCCAC.

GACGTT-3′; HCMV *pp150*-forward 5′- GGTTTCTGGCTCGTGGATGTCG-3′ and reverse 5′- CACACAACACCGTCGTCCGATTAC-3′; *GAPDH*-forward 5′-CTGTTG.

CTGTAGCCAAATTCGT-3′ and reverse5′-ACCCACTCCTCCACCTTTGAC-3′. The qPCR procedure was as follows: 3 min at 95 °C for initial denaturation, 40 cycles of 5 s at 95 °C and 30 s at 60 °C. Each group was assayed in triplicates (n = 3). The difference in HCMV DNA copy number between the PTE treatment group and the HCMV infection alone group was calculated using the 2^−△△Ct^ method. First, the housekeeping gene GAPDH was used to normalize the Ct values of all treatment and control samples, i.e., ΔCt = Ct (sample) − Ct (GAPDH). Second, the Ct values of the PTE treatment groups were compared to those of the control samples. ΔΔCt = ΔCt (sample) − ΔCt (control sample). The relative expression of the target gene is represented by 2^−△△Ct^.

### Indirect immunofluorescence assay (IFA)

IFA was carried out in a 48-well plate, and PTE or FOS treatment and HCMV infection were the same as above. The cell samples were fixed for 30 min using precooled acetone/methanol (v/v, 1:1) at − 20 °C. Then, the fixed samples were incubated with 5% nonfat dry milk for blocking for 1 h at room temperature. After five washes with PBS, the cell samples were treated with HCMV IE1/2 mouse monoclonal antibody overnight at 4 °C, followed by incubation with secondary antibody for 1 h at room temperature. The fluorescence signal was observed using a Zeiss microscope (Axio Vert.A1).

### Tissue culture infective dose (TCID50) assay and half-maximal inhibitory concentration (IC50) measurements

TCID_50_ assays were performed in 96-well plates. When WI-38 cells grew to 80% confluence, they formed a monolayer. HCMV samples were serially diluted tenfold. Subsequently, an aliquot of 100 μL from each dilution was inoculated into WI-38 cells and maintained at 37 °C in a 5% CO_2_ incubator (Heraeus Instruments). The experiment was performed simultaneously in 8 replicates per dilution. The cytopathic effect (CPE) was monitored and recorded daily until the CPE wells did not change. Finally, the virus titre was calculated according to the classical Reed-Muench method [31, 32]. The falf-maximal inhibitory concentration (IC50) was determined based on the results of the TCID50 assay according to nonlinear trajectory analysis using GraphPad Prism.

### Senescence-associated beta-galactosidase (SA-β-gal) staining

The level of cellular senescence was assessed by an SA-β-gal staining kit essentially as described in a previously published paper [30]. Briefly, WI-38 cells were cultured in 6-well plates with different drug-treatments until the indicated time. Then, the cells were washed three times with PBS and fixed for 5 min in 0.25% glutaraldehyde. After this, the cells were incubated with SA-β-gal staining solution at 37 °C for 3–16 h. Finally, the fields of SA-β-gal-positive cells (blue-green cells) were randomly selected and then counted, and the percentage of senescent cells was evaluated from three independent experiments.

### Measurement of intracellular reactive oxygen species (ROS)

The ROS assay was performed in 6-well plates, and the cell samples with different treatments were the same as those shown above. The intracellular ROS level was calculated with an ROS Assay Kit according to the manufacturer’s instructions. First, the ROS probe 2',7'-dichlorodihydrofluorescein diacetate (H_2_DCFDA) was diluted with FBS-free DMEM to a final concentration of 10 μM. Then, the cells were washed twice with PBS and treated with H_2_DCFDA for 20–30 min at 37 °C. Then, the cells were washed three times with FBS-free DMEM. Finally, the cells were digested with trypsin and collected to measure the intracellular fluorescence intensity by flow cytometry.

### Statistical analysis

Data are expressed as the mean ± standard deviation (SD), and significant differences between the different groups were determined by Student’s t test. The differences were considered statistically significant at *P* < 0.05.

## Results

### Effect of PTE on WI-38 cell viability

To explore the effects of PTE on the viability of WI-38 cells, we conducted a CCK-8 assay to analyse the potential cytotoxicity of PTE. The result was shown in Fig. 1B, C, no obvious cytotoxicity was detected after 5 days of treatment in the concentration range of 1 to 30 μM (*P* > 0.05); however, when the concentration reached 40 μM or higher, compared with the control, PTE showed obvious cytotoxicity, and the difference was extremely significant (*P* < 0.001). Thus, the half-maximal cytotoxicity concentration (CC50) of PTE treatment was 35.15 μM in WI-38 cells (Fig. 1C). Thus, we chose 10 μM as the concentration for the next study unless specifically indicated. Moreover, the cells treated with 5 μM, 10 μM or 20 μM PTE showed normal fibrous morphological features (Fig. 1D, upper panel).

### Antiviral activity of PTE on HCMV

Previously, we observed obvious morphological changes and cytopathic effects (CPE) in HCMV-infected cells [30, 31]. As shown in Fig. 1D, WI-38 cells infected with HCMV alone at an MOI of 0.5 displayed typical CPE characterized by cell rounding and enlargement with inclusion bodies at 5 dpi, while pretreatment of the cells with 5 μM PTE significantly reduced the degree of CPE, and the morphology of WI-38 cells treated with 10 μM and 20 μM was similar to that of the mock-infected group. These results indicated that PTE could protect the morphological features of the WI-38 cells infected with HCMV and further suggested that PTE might have anti-HCMV activity.

**Fig. 2:**
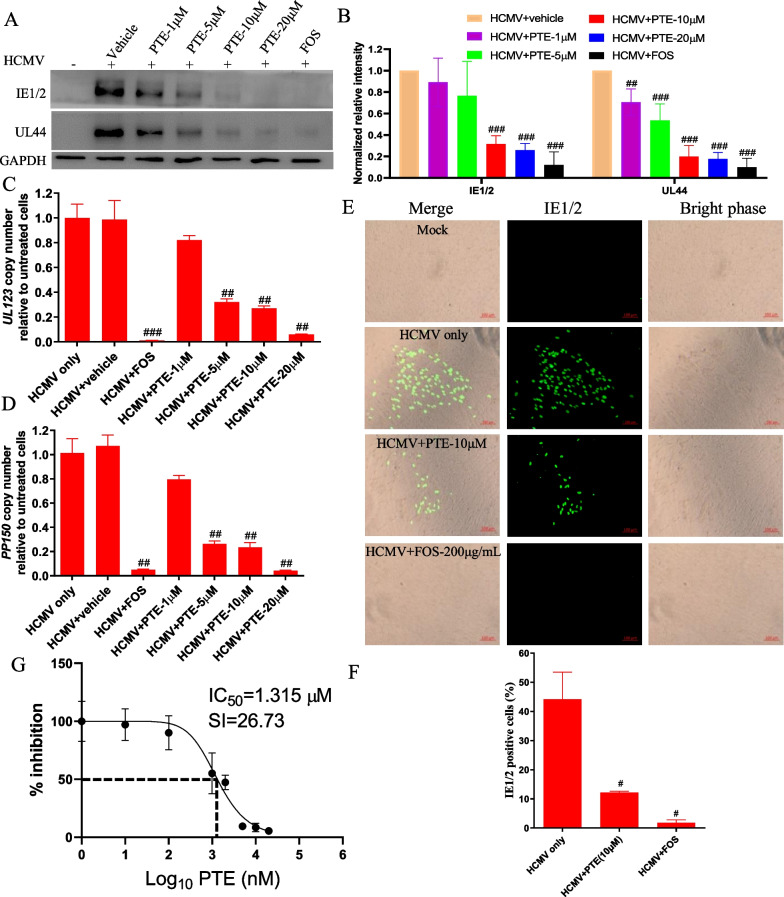
Antiviral activity of PTE on HCMV. **A** Expression levels of IE1/2 and UL44 in the HCMV-infected cells with or without PTE treatment. WI-38 cells were pretreated with PTE (1 μM, 5 μM, 10 μM, 20 μM) for 2 h before HCMV (MOI = 0.5) inoculation. Protein expression intensity was analysed using western blotting at 5 dpi. GAPDH was used as the loading control. Representative images were acquired from three different independent experiments. **B** Quantitative analysis of the protein levels of IE1/2 and UL44 from three independent experiments. **C** and **D** Effects of PTE on viral *UL123* and *PP150* copy number in HCMV-infected cells (MOI = 0.5). The HCMV DNA was extracted 5 dpi as mentioned in the Materials and Methods section. The copy numbers of *UL123* and *PP150* gene were measured with the 2^−△△Ct^ method, the HCMV infection only group was used as control. **E** and **F** IFA results in the HCMV-infected (MOI = 0.5) WI-38 cells with or without PTE treatment. HCMV inoculation and PTE treatment were performed as described in the Materials and Methods section. The IE1/2-positive signals were detected by a mouse IE1/2 monoclonal antibody combined with FITC-conjugated goat anti-mouse IgG. **G** PTE inhibited the production of infectious virions. WI-38 cells were pretreated with the indicated concentrations of PTE for 2 h and then infected with HCMV (MOI = 0.5). The cell culture supernatant samples were collected at 5 dpi. The virus titre was calculated by TCID_50_ assays according to the Reed-Muench method. The falf-maximal inhibitory concentration (IC50) was determined based on the results of the TCID50 assay according to nonlinear trajectory analysis using GraphPad Prism. ^#^*P* < 0.05, ^##^*P* < 0.01, ^###^*P* < 0.001

To verify the anti-HCMV activity of PTE, we performed Western blotting, qPCR, IFA and TCID50 assays to assess viral protein expression, viral gene copy number and virus titre. First, we evaluated the protein expression levels of HCMV IE1/2 and UL44. Compared with those of the vehicle control group, the expression levels of the IE1/2 and UL44 proteins were reduced rapidly after treatment with PTE at the indicated concentrtions (Fig. 2A, B). The results were consistent with the *UL123* and *PP150* copy numbers shown in Fig. 2C, D. The *UL123* and *PP150* copy numbers decreased rapidly at 5 μM or higher (*P* < 0.01) compared with those of the HCMV infection group. The results indicated that PTE inhibited HCMV DNA replication.

Second, we carried out IFA to further assess the anti-HCMV activity of PTE on WI-38 cells (Fig. 2E, F). Similar to the results of published papers [30, 31], the IE1/2 positive signal was concentrated in the nuclei of the HCMV-infected cells. Compared with that of the HCMV-infected alone group, the number of IE1/2-positive cells decreased dramatically when the cells were treated with PTE (10 μM).

Finally, a TCID50 assay was conducted to further investigate the changes in viral titres induced by PTE treatment. The result was showed in Fig. 2G. PTE inhibited the production of HCMV progeny virions in a dose-dependent manner at the indicated concentrations (0.01 μM, 0.1 μM, 1 μM, 2 μM, 5 μM, 10 μM, 20 μM), and the half-maximal inhibitory concentration (IC50) was determined to be 1.315 μM. The selectivity index (SI) of PTE was calculated as 26.73. In conclusion, PTE could suppress HCMV infection in host WI-38 cells with respect to protein expression, *UL123* and *PP150* gene expression and viral titres.

### PTE inhibited HCMV at a stage post-virus entry

**Fig. 3:**
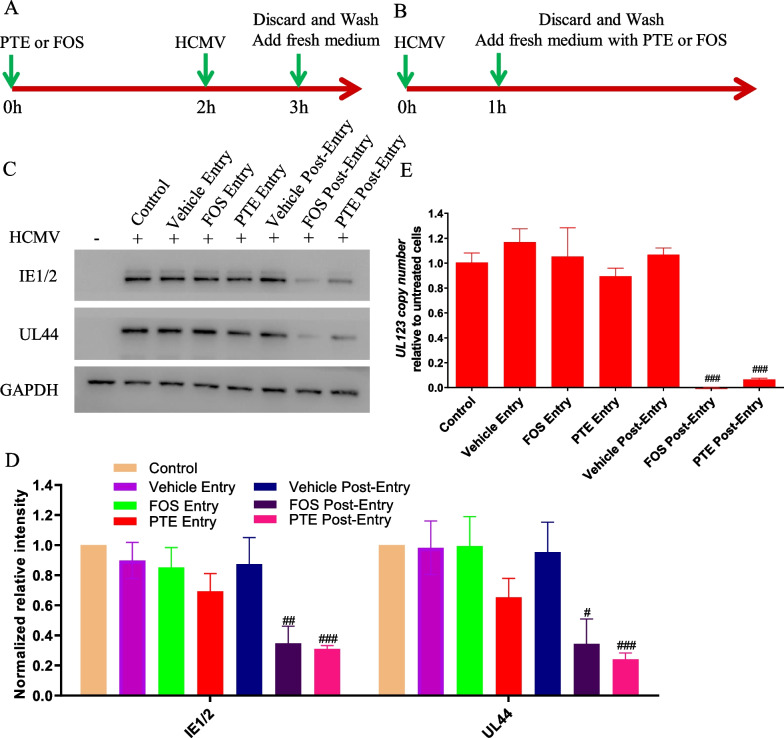
PTE inhibited HCMV at a stage post virus entry. **A**, **B** Schematic representation of the Time-of-addition experimental design. **A** For Entry treatment, PTE or FOS was added to WI-38 cells for 2 h before HCMV inoculation, and at 1 hpi, the virus-drug mixture was discarded, and fresh culture medium was added. **B** For Post-Entry treatment, after 1 h of HCMV inoculation and viral attachment, the culture medium containing virus was replaced with fresh culture medium with PTE or FOS. For all the experimental groups, WI-38 cells were infected with HCMV at an MOI of 5 and collected at 3 dpi, and the concentrations of PTE and FOS used here were 10 μM and 200 μg/mL, respectively. HCMV proteins and DNA copy number in these groups were detected and quantified using Western blotting analysis (**C**, **D**) and qPCR (**E**). GAPDH was used as the loading control. Representative images were acquired from three independent experiments, and data obtained from three independent experiments were used for quantitative analysis. The HCMV infection alone group was used as the control. ^#^*P* < 0.05, ^##^*P* < 0.01, ^###^*P* < 0.001

To determine at what point during viral infection PTE mediated its inhibitory effect, we examined the expression level of viral protein and the *UL123* gene using Entry (Fig. 3A) and Post-Entry assays (Fig. 3B) at 3 dpi. In the Entry assay group, WI-38 cells were pretreated with PTE before HCMV attachment and entry. There was no significant change in the expression levels of IE1/2 and UL44 compared to those of the control group (*P* > 0.05). However, we observed that the viral protein decreased dramatically when HCMV was first allowed to enter the cell and then subjected to PTE treatment. The inhibitory effect was similar to that in the FOS-treated sample, as FOS mainly targets DNA polymerase, which affects the HCMV cycle of the replication period [8]. The results suggested that PTE predominantly acts after virus entry and membrane fusion (Fig. 3C, D). Moreover, the copy number of *UL123* in all the above groups was calculated to verify this result. Similar to viral protein level results, the copy number of *UL123* decreased significantly only in Post-Entry assay (Fig. 3E) (*P* < 0.001).

### PTE suppressed HCMV-induced cell senescence

**Fig. 4:**
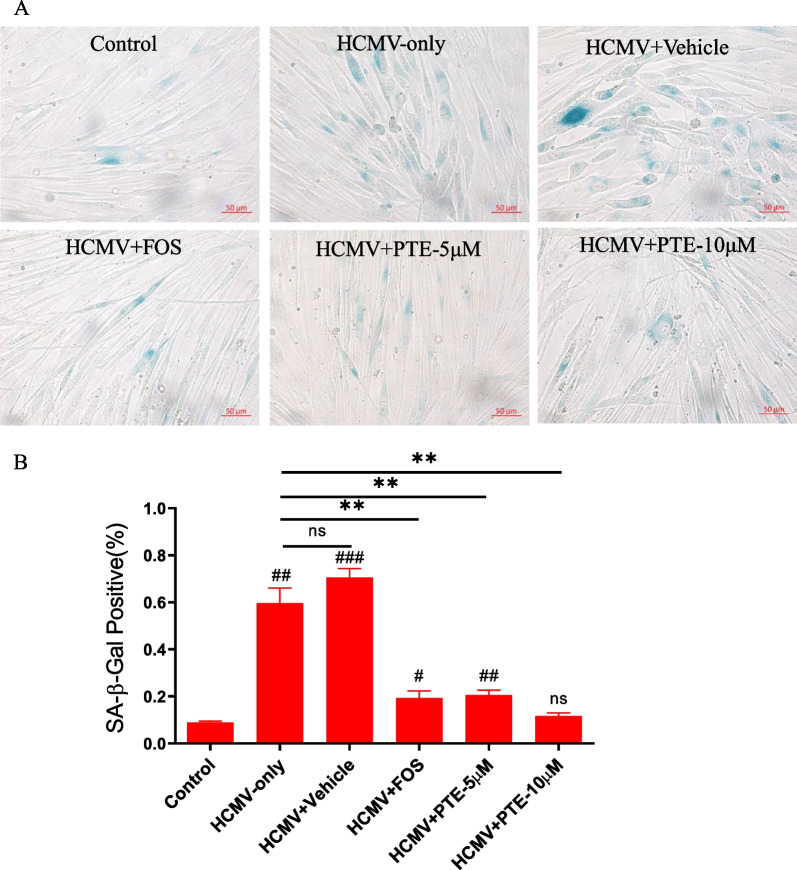
Effect of PTE on SA-β-Gal staining in HCMV-infected WI-38 cells. Before virus infection, the culture medium was replaced with 0.2% FBS for 48 h to synchronize cells in the G_0_ phase. Then, serum-starved WI-38 cells were infected with HCMV (MOI = 0.5) with or without PTE treatment (5 μM, 10 μM) at 5 dpi and submitted for SA-β-Gal staining according to the Kit instructions. (A) Representative images were selected from three independent experiments. (B) Quantitative analysis of SA-β-gal-positive cells out of the total cells from the average data obtained from three independent experiments. The sample without HCMV infection was used as a control. ^#^*P* < 0.05 versus the control, ^##^*P* < 0.01 versus the control, ^###^*P* < 0.001 versus the control. **P < 0.01 versus the HCMV-only group

WI-38 cells are also widely used as a model of cell senescence, and HCMV infection could induce cellular senescence in previous studies [29, 30, 33, 34]. The accumulation of a lysosomal enzyme termed SA-β-Gal was the first and is still the most widely used biomarker to detect senescent cells in cultured cells; hence, we performed classical SA-β-Gal staining to determine whether HCMV infection could promote the progression of cell senescence in young WI-38 cells (PD30). As shown in Fig. 4, HCMV infection (HCMV infection alone or with vehicle treatment) at an MOI of 0.5 could induce 60–80% cell senescence at 5 dpi, and this result was similar to previous studies [29, 30]. While the percentage of SA-β-Gal-positive cells decreased to 20% and 10% after treatment with 5 μM and 10 μM PTE, respectively, the effect of 10 μM PTE was the same as that of the control group (*P* > 0.05). Compared to that of the HCMV-alone group, the number of SA-β-Gal-positive cells in the two treatment groups with different concentrations of PTE (5 μM, 10 μM) showed significant differences (*P* < 0.01). These results indicated that PTE significantly suppressed the senescence phenotype in WI-38 cells infected with HCMV.

### PTE suppressed HCMV-induced activation of molecular mechanisms of senescence

**Fig. 5:**
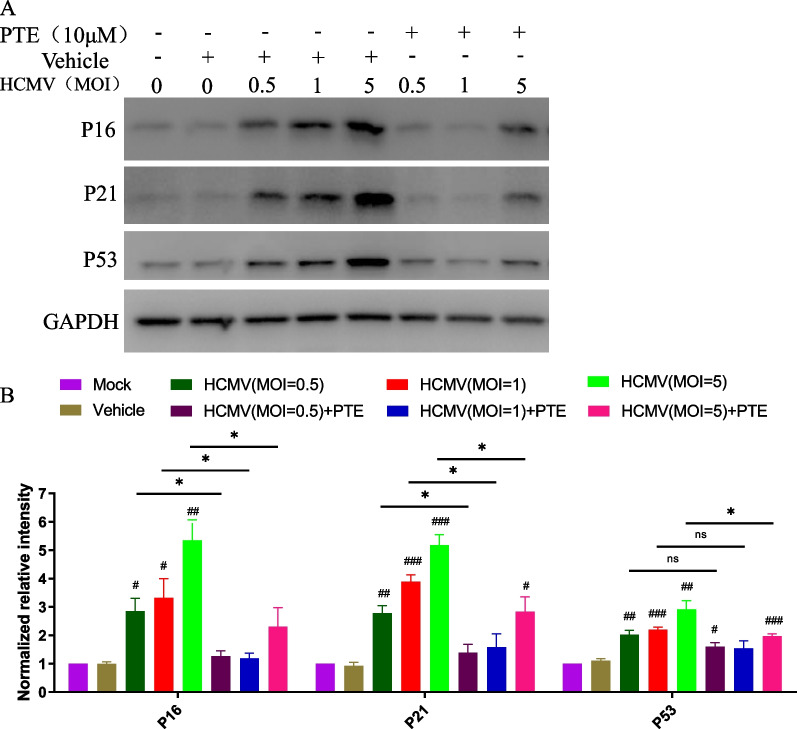
Effects of PTE on HCMV-induced expression of p16, p21 and p53. **A** Serum-starved WI-38 cells were infected with the indicated MOI of HCMV, and the samples were harvested at 5 dpi to analyse the expression levels of the above molecules using Western blotting. GAPDH was used as the loading control. Representative images shown here were selected from three independent experiments. **B** Quantitative analysis of the protein levels of p16, p21 and p53. The sample without HCMV infection was used as the mock control, ^#^*P* < 0.05 versus the mock control, ^##^*P* < 0.01 versus the mock control, ^###^*P* < 0.001 versus the mock control. *P < 0.05 versus the HCMV + PTE group

In addition to the increasing number of SA-β-Gal-positive cells, the accumulation of p16 and p21 is another characteristic of senescent cells and was also used as a biomarker of cellular senescence. p16 and p21 are two cyclin-dependent kinase inhibitors that are components of tumour suppressor pathways governed by p53, and their expression levels play critical roles in the establishment and maintenance of senescence-associated growth arrest [35]. We then evaluated the expression levels of p16, p21 and p53 in the HCMV-infected cells of different MOIs with or without PTE treatment. We found that HCMV infection increased the expression levels of all three proteins in an MOI-dependent manner, and this result was in accordance with previous studies [29, 30]. However, PTE treatment rapidly alleviated this effect (Fig. 5). These results suggested that PTE had a significant inhibitory effect on HCMV-induced expression of p16, p21 and p53.

### PTE suppressed HCMV-induced ROS production

**Fig. 6:**
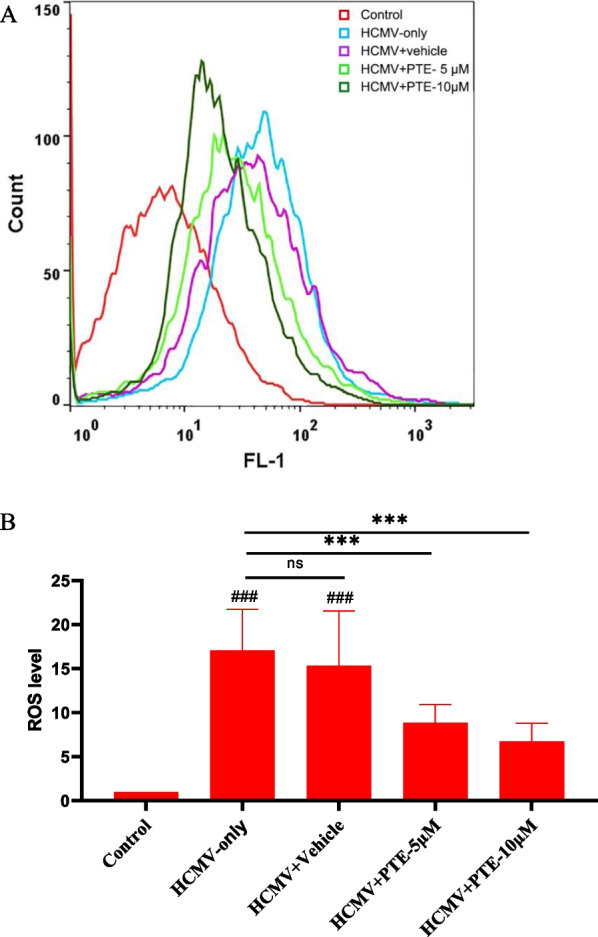
Effects of PTE on HCMV-induced ROS production. WI-38 cells were infected with HCMV (MOI = 0.5) with or without treatment with PTE (5 μM, 10 μM), and the samples were subjected to ROS measurement at 5 dpi using the probe H_2_DCFDA by flow cytometry. **A** Representative results of ROS production detected by Flow cytometry. **B** Quantitative analysis of the ROS levels obtained from the data of three independent experiments. A mock-infected sample was used as a control. ^###^*P* < 0.001 versus the control. ***P < 0.001 versus the HCMV-only group

Oxidative stress is closely related to cell senescence and might function as a common trigger for activation of the senescence programme [36, 37]. Oxidative stress is also involved in the process of viral infection [38]. Our previous studies found that HCMV infection induced a sharp rise in ROS production in young WI-38 cells [29, 30]. Hence, we also analysed the effects of PTE on HCMV-induced ROS production. As shown in Fig. 6, HCMV infection elevated intercellular ROS production, while PTE treatment significantly alleviate this effect (*P* < 0.001). This result suggested that PTE could suppress HCMV-induced ROS production.

## Discussion

In this study, we report potent suppressive effects of PTE on HCMV infection in WI-38 cells for the first time, and the inhibitory effect mainly occurs during the Post-Entry period. Further study demonstrated that the anti-HCMV effect was partly related to the inhibition of HCMV-induced cell senescence and the activation of the molecular mechanism of ROS production.

As RES has multiple cellular targets, the modulatory effects of RES on viral infection and pathogenesis may be mediated through diverse mechanisms. Current studies have shown that RES could have an antiviral effect on the entry, replication, and transcription stages in different viruses [23]. Structurally, PTE is a dimethyl ether and metabolically more stable analogue of RES (Fig. 1A), and thus, PTE increases lipophilicity and restricts glucuronidation and sulfation, hence improving the solubility, absorption, and bioavailability compared to RES. Thus, PTE was found to be more effective in controlling SARS-CoV-2 replication than RES at low concentrations [26]. The HCMV gene is known to be expressed in three temporal cascades, designated the immediately early, early and late stages. Among the three phases, IE1-72 kDa and IE2-86 kDa, which belong to nuclear phosphoproteins, are the major IE protein species. They are key transcriptional regulators that are well known for activating viral expression to facilitate productive infection [39]. Moreover, the protein encoded by the early viral gene (UL44) is necessary for viral DNA replication [40]. Therefore, we evaluated the anti-HCMV activity of PTE by the measurement of immediately early (IE1/2) and early (UL44) proteins and *UL123* and *PP150* gene copies, and the data in this study revealed that all of them were significantly decreased (Fig. 2A–D). In addition, the IFA and TCID_50_ assays are also well proved the anti-HCMV effect of PTE (Fig. 2E–G). These results demonstrate that PTE possesses anti-HCMV activity in vitro.

Although replication of different viruses varies, there are several common stages in the life cycle of viruses, including adsorption, entry, gene transcription, protein translation, assembly, and release, which are all desirable antiviral targets [41]. As a result, identifying the stage at which a drug works is important for antiviral research. The time-of-addition experiments shown in Fig. 3 were used to explain this issue. A significant reduction in viral proteins and *UL123* gene copies was observed when PTE was added after removal of the virus inoculum, and the result was similar to that of FOS, which exerts its anti-HCMV effect during the replication and translation periods [8]. Treatment with PTE before HCMV entrance did not result in anti-HCMV activity, although HCMV and PTE coexisted for 1 h. The expression levels of viral proteins and *UL123* gene copies both indicated the Post-Entry stage as the target period when PTE takes effect, and PTE does not interfere with virus adsorption, entry and membrane fusion.

An increasing number of studies show that virus-induced senescence (VIS), including that in SARS-CoV-2 infection [42], is a universal phenomenon, and excessive accumulation and persistence of senescent cells can become detrimental and promote pathology and dysfunction [43]. In HCMV, IE1 protein activates and interacts with p53 and then induces the accumulation of p53 [44, 45]. The IE2 protein inhibits cellular DNA synthesis, resulting in cell cycle arrest through a functional p53 pathway [46]. The interaction of IE1, IE2 and p53 above ultimately evokes the senescence phenotype in HCMV-infected cells [33, 45, 46]. In addition, the expression of p16 is upregulated by HCMV infection and required for optimal viral replication [34]. The findings that HCMV triggers p16 in the early phases of infection and does not replicate in cells lacking a functional p16, therefore, demonstrate that HCMV may exploit the p16-pRb axis to stimulate the senescence program favorable for its replication [34]. As HCMV infection is effectuated in arrested cells, the observed phenotype, characterized by enlargement, flattening and irregularity, is ascribed to the activation of the molecular mechanism of cellular senescence. PTE in this study could alleviate the increasing SA-β-Gal-positive cells and specific markers of senescence induced by HCMV infection (Figs. 4 and 5). The results demonstrate that the inhibitory effects of PTE on HCMV infection are partly related to the molecular mechanisms of senescence, and suggest that senolytic targeting of virus-infected cells may be a treatment option against viral infections.

Oxidative stress has always been one of the major factors of cellular senescence; either oxidizing products of cell metabolism (e.g., ROS) or known oxidative agents (e.g., H_2_O_2_) induce cellular senescence, and reducing ROS production protects the cells from the effects of the cellular senescence process [47]. Moreover, ROS enhance HCMV infection, and this effect could be partly suppressed by N-acetylcysteine, a common H_2_O_2_ scavenger [48]. In this study, we observed that HCMV infection increased ROS production, while PTE treatment reduced this effect. Thus, we propose that the antioxidant activity of PTE may be responsible for the suppression of HCMV infection and alleviation of cellular senescence. In conclusion, decreased p16 expression and ROS production caused by PTE treatment in HCMV-infected cells would partly in turn affect the subsequent proliferation of the virus and thus exert an antiviral effect.

This study has limitations. First, all of the findings here are made in WI-38 cells, a widely used human fibroblast cell line, and have not been generalized to other host cell types that HCMV is known to infect (e.g., dendritic cells, monocytes and endothelial cells). Second, we only performed this study with the HCMV Towne strain. HCMV varies frequently among lab strains, clinical strains and clinical variants. Without a doubt, these two in-depth studies are worth performing. Overall, this study indicates that PTE has strong anti-HCMV activity in vitro, and the mechanism has been partially shown to involve the suppressive effect of the molecular mechanisms of cellular senescence and ROS production induced by HCMV. Given the lack of vaccines, this study provides a solid theoretical basis for the development of PTE as an effective anti-HCMV drug.

## Data Availability

The datasets used and/or analysed during the current study are available from the corresponding author on reasonable request.

## Abbreviations

HCMV: Human cytomegalovirus
PTE: Pterostilbene
RES: Resveratrol
CDV: Cidofovir
GCV: Ganciclovir
FOS: Foscarnet
FBS: Fetal bovine serum
ROS: Reactive oxygen species
PD: Population doubling
MOI: Multiplicity of infection
SA-β-Gal: Senescence-associated β-galactosidase
H_2_DCFDA: 2',7'-Dichlorodihydrofluorescein diacetate
PVDF: Polyvinylidene fluoride
qPCR: Quantitative polymerase chain reaction
Ct: Cycle threshold
IFA: Indirect immunofluorescence assay
CPE: Cytopathic effect
TCID50: Tissue culture infective dose
CC50: Falf-maximal cytotoxicity concentration
IC50: Falf-maximal inhibitory concentration
SI: Selectivity index
VIS: Virus-induced senescence
